# Visual Dysfunction in Diabetes

**DOI:** 10.1146/annurev-vision-111022-123810

**Published:** 2023-05-10

**Authors:** Erika D. Eggers

**Affiliations:** Departments of Physiology and Biomedical Engineering, University of Arizona, Tucson, Arizona, USA

**Keywords:** retina, human, rodent, mouse, rat, diabetes, neurons, physiology

## Abstract

Although diabetic retinopathy (DR) is clinically diagnosed as a vascular disease, many studies find retinal neuronal and visual dysfunction before the onset of vascular DR. This suggests that DR should be viewed as a neurovascular disease. Prior to the onset of DR, human patients have compromised electroretinograms that indicate a disruption of normal function, particularly in the inner retina.They also exhibit reduced contrast sensitivity. These early changes, especially those due to dysfunction in the inner retina, are also seen in rodent models of diabetes in the early stages of the disease. Rodent models of diabetes exhibit several neuronal mechanisms, such as reduced evoked GABA release, increased excitatory glutamate signaling, and reduced dopamine signaling, that suggest specific neuronal deficits. This suggests that understanding neuronal deficits may lead to early diabetes treatments to ameliorate neuronal dysfunction.

## INTRODUCTION

Diabetic eye disease is of increasing concern due to the increasing prevalence of diabetes mellitus globally (NCD Risk Factor Collab. 2016). Approximately 35% of people with diabetes develop some form of diabetic retinopathy (DR), and approximately 10% develop severe, sight-threatening forms of DR such as retinal vascular proliferation, retinal detachment, and macular edema (Yau et al. 2012). Clinically, the presence of DR is diagnosed from low stage to high stage using retinal fundus photographs to identify the presence of exudates, hemorrhages, microaneurysms, microvascular abnormalities, neovascularization (Early Treat. Diabet. Retin. Study Res. Group 1991), and macular edema (Wilkinson et al. 2003). The DR retinal vascular problems of diabetes can be characterized as late-stage problems, since they typically take 10–15 years to develop (Yau et al. 2012). However, significant evidence exists that there are early neuronal deficits before the development of DR. Visual and retinal structural changes appear prior to the late-stage vascular problems of DR in both humans with diabetes and animal models of diabetes (Lynch & Abramoff 2017). Changes in contrast sensitivity and electroretinogram (ERG) activity, some as early as 4 weeks after diabetes induction in rodent models (Aung et al. 2013, Layton et al. 2007), suggest early neuronal damage. Many studies have also shown thinning of retinal layers before DR begins. Thus, diabetic eye disease is now understood as both a vascular and a neuronal disease of the retina (Jackson & Barber 2010).

**Figure 1:**
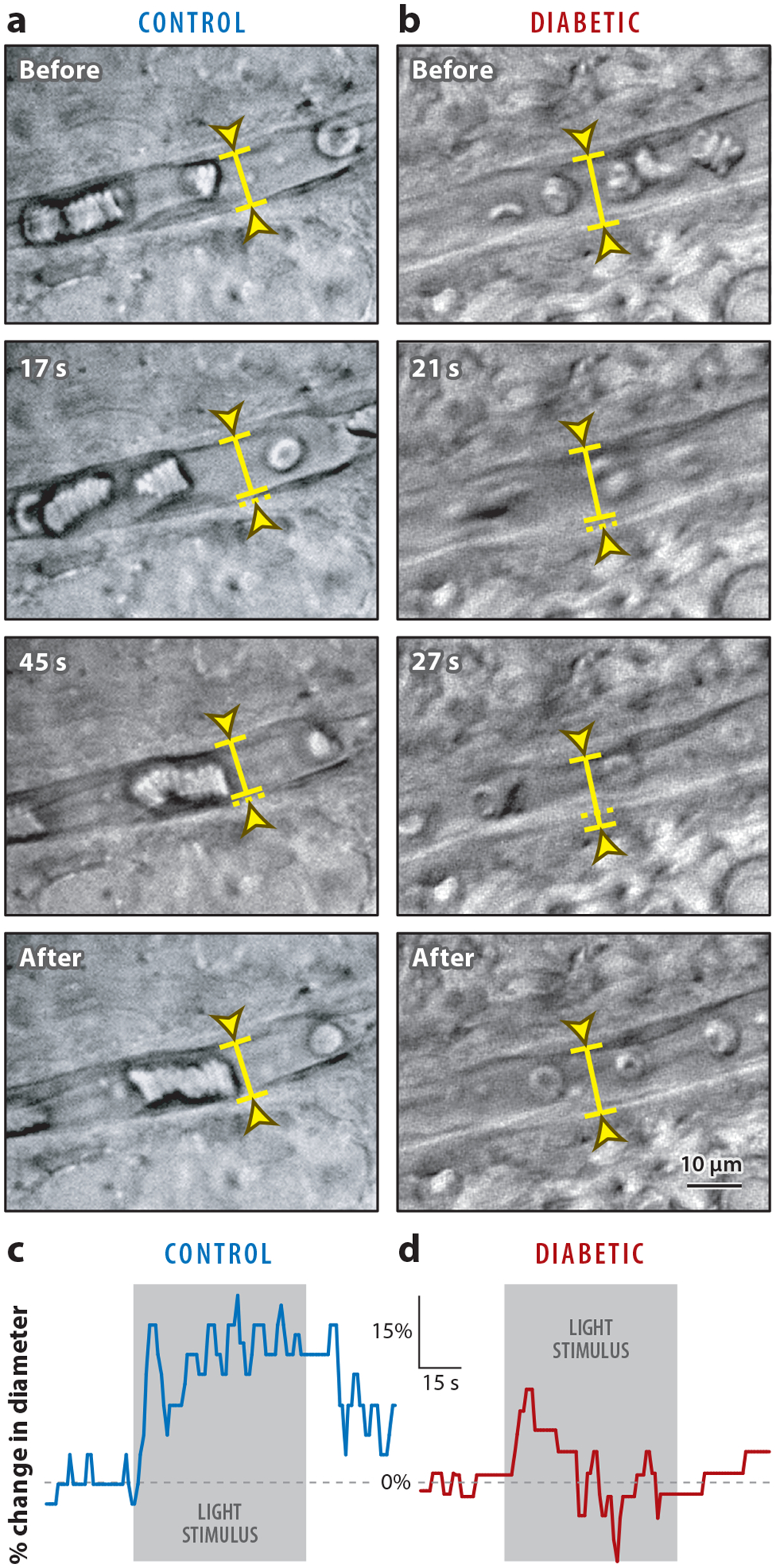
Light-evoked vasodilation is reduced in diabetic retinas. (*a*,*b*) Infrared–differential interference contrast (IR-DIC) images of the vitreal surface of the retina, illustrating the light-evoked responses of small arterioles. (*a*) In a control retina, light stimulation evokes a large vasodilation (at 17 and 45 s after onset of the light stimulus). (*b*) In a diabetic retina, light evokes a smaller dilation (at 21 s), followed by a constriction (at 27 s). The diameters of both control and diabetic vessels recover to baseline after light stimulation ends. (*c*,*d*) Light-evoked arteriole dilation in (*c*) a normal and (*d*) a diabetic retina. Light stimulation evokes a smaller dilation, followed by a constriction, in the diabetic retina. Figure adapted with permission from Mishra & Newman (2010).

Previous reports have also suggested that changes in retinal activity may be correlated with pre-DR changes and/or later DR changes in retinal vasculature. An example of this connection is the retinal hyperemia response, which is the dilation of retinal blood vessels in response to neuronal stimulation with light. Retinal hyperemia is compromised in both diabetic humans with no DR (Garhofer et al. 2020, Pemp et al. 2009) and animal models of diabetes (Mishra & Newman 2010, 2011) (Figure 1). The significant visual deficits in early diabetes and the potential connection of retinal neuronal light responses and vascular function suggest that understanding how retinal function and vision changes in early diabetes is vital. Since changes in retinal function and vision happen before clinically detectable DR, these changes have important potential both for screening for diabetic eye damage and as indicators for potential early treatments. This review focuses on the evidence and mechanisms for early diabetic damage to retinal neurons before DR develops.

## VISUAL DYSFUNCTION IN EARLY DIABETES

**Figure 2:**
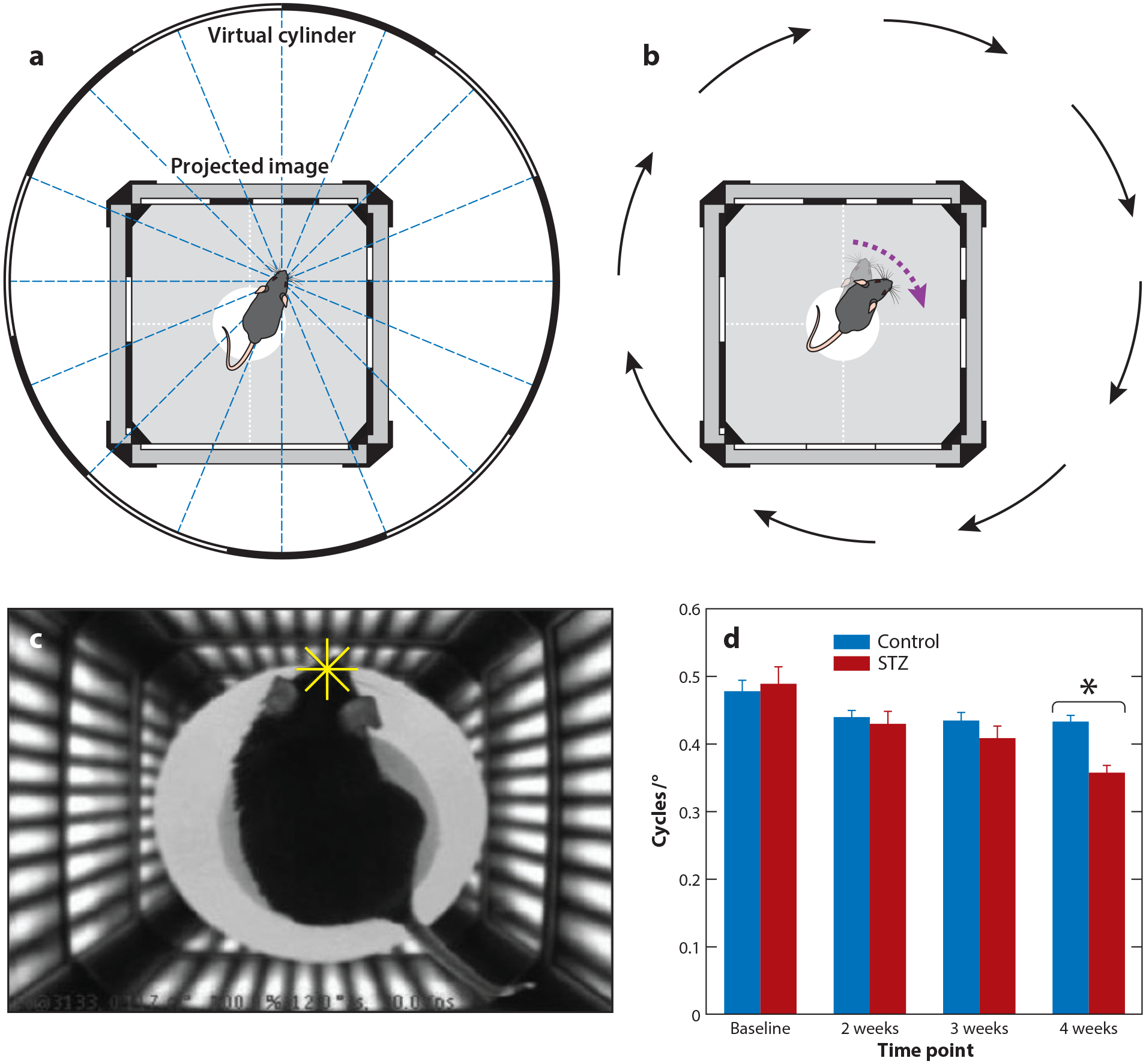
Rodent visual testing in diabetes. (*a*) A virtual cylinder is projected in 3D coordinate space on the monitors. The head of the mouse centers the rotation of the cylinder due to the optomotor response. (*b*) When the cylinder is rotated, the mouse tracks the drifting grating with head and neck movements. (*c*) A single-frame video camera image of a mouse tracking the cylinder grating. The four-line cross-hair (*yellow*) is positioned between the eyes of the mouse, and the coordinates are used to center the rotation of the cylinder. (*d*) Changes in spatial frequency thresholds after 1 month of diabetes. Optokinetic response tracking in rats before the induction of diabetes and after 2, 3, and 4 weeks of hyperglycemia (*blue bars*) is compared with a control (*red bars*). Data are the average of two independent experiments with at least four rats per group (*n* ≥ 8). Error bars denote SD. *p < 0.05 compared with control using Student’s t-test. Panels *a–c* adapted with permission from Prusky et al. (2004). Panel *d* adapted with permission from Kirwin et al. (2011). Abbreviations: SD, standard deviation; STZ, streptozotocin.

Visual dysfunction in early diabetes has been seen in both contrast sensitivity—the ability to differentiate an image from a background—and visual acuity—the ability to see small objects clearly. In humans, these are typically measured by verbal feedback, but animal models are more difficult. In rodents, both acuity and contrast sensitivity are measured using the optokinetic response, where animals turn their head to follow moving bars (Prusky et al. 2004) (Figure 2). The contrast and size of the bars are varied to find the minimal contrast and bar size that can be sensed by the animals. This allows an estimate of visual acuity and contrast sensitivity in animal models of diabetes.

There are several animal models of diabetes that have been used to determine how early diabetes affects visual function (Olivares et al. 2017). The most commonly used is the streptozotocin (STZ) model. This model uses injections of STZ, which is taken up by glucose transporters in pancreatic beta cells. This causes beta cell death and leads to hyperglycemia, making it a type 1 diabetes model that can be used in many species (Furman 2015, Kolb 1987). Animals injected with STZ typically have hyperglycemia within a few days of injections, making the timing of this model easily controlled. Another commonly used model is the Ins2^Akita^ mouse line, which has a mutation in the *insulin2* gene that causes accumulation in the endoplasmic reticulum of pancreatic beta cells. This ultimately causes beta cell death and gradual hyperglycemia beginning at approximately 4 weeks of age (Barber et al. 2005).

**Figure 3:**
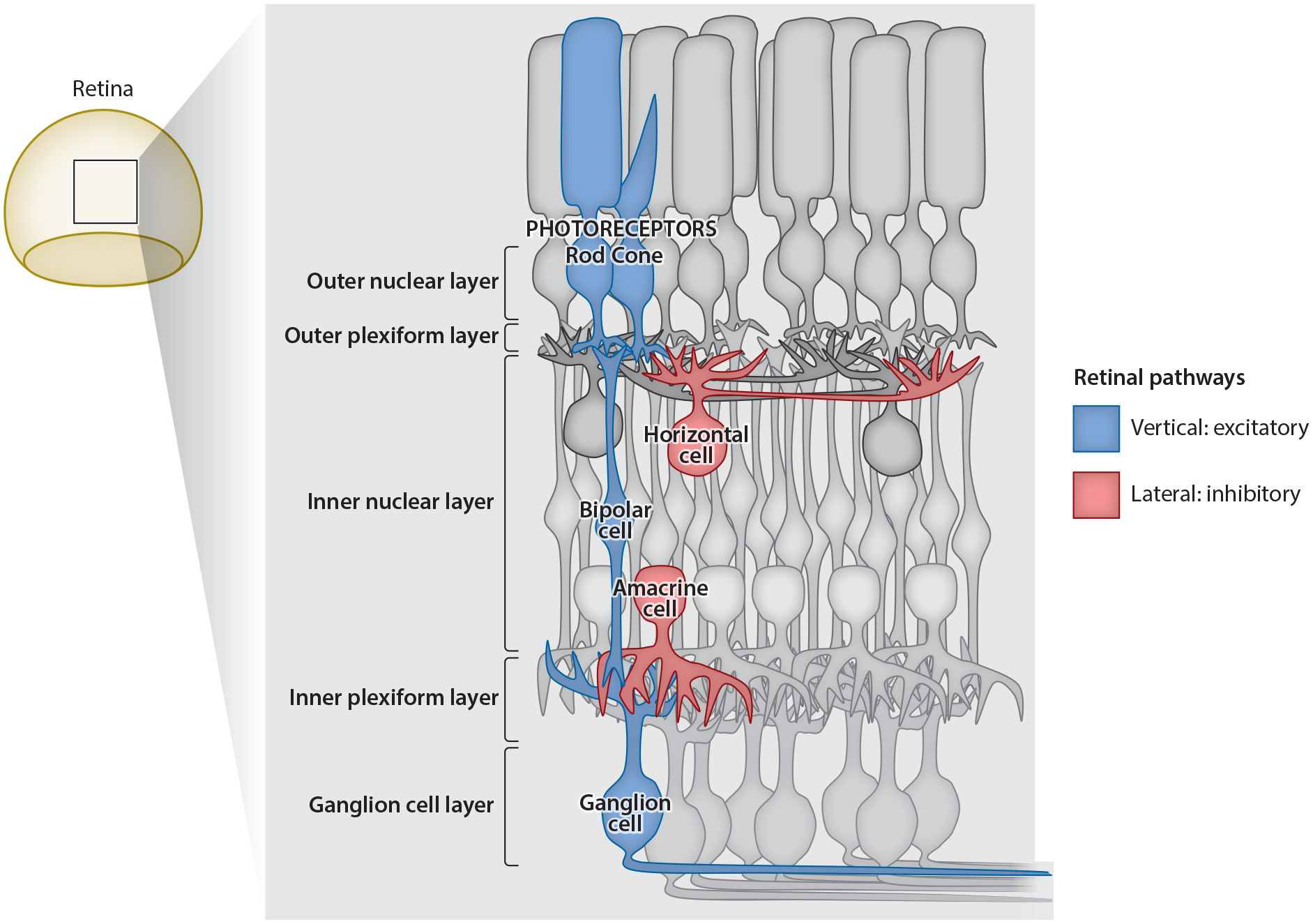
Retinal signaling pathways. The excitatory retinal pathway (*blue*) starts with rod and cone photoreceptors that send excitatory input to bipolar cells that relay information to ganglion cells, which are the output neurons of the retina. The inhibitory retinal pathway (*red*) consists of horizontal cells, which send feedback to photoreceptors and bipolar cells, and amacrine cells, which send feedback to bipolar cells and ganglion cells.

Both type 1 (Di Leo et al. 1992, Harris et al. 1996, Lopes de Faria et al. 2001) and type 2 (Dosso et al. 1996) diabetic humans with no DR exhibited reduced contrast sensitivity. Decreases in contrast sensitivity were correlated with worse glycemic control, measured by higher glycosylated hemoglobin (HbA1c) levels that reflect recent blood sugar levels (Di Leo et al. 1992, Pramanik et al. 2020). Contrast sensitivity was decreased at mesopic (rod- and cone-mediated) and low photopic (cone-mediated) intensities (Dosso et al. 1996), but general changes in visual acuity have not typically been observed. Changes in the timing of visually evoked potentials that measure activity in primary visual cortex appear within 6 months after diagnosis of diabetes (Parisi et al. 1997, Uccioli et al.1995). Rodent models exhibit decreases in contrast sensitivity and visual acuity, measured by the optokinetic response, as early as 4 weeks after induction of diabetes with STZ (Aung et al. 2014, Kirwin et al. 2011, Miller et al. 2018) (Figure 2d). Together, these results suggest that early diabetes functionally affects the inner retina (inner nuclear layer, inner plexiform layer, and ganglion cell layer; see Figure 3), which dominates these contrast sensitivity measurements, and that visual changes in human diabetic patients are mirrored by those seen in animal models.

## RETINAL NEURONAL DYSFUNCTION AND DEATH IN EARLY DIABETES

### Changes in the Retinal Electroretinogram in Diabetes

**Figure 4:**
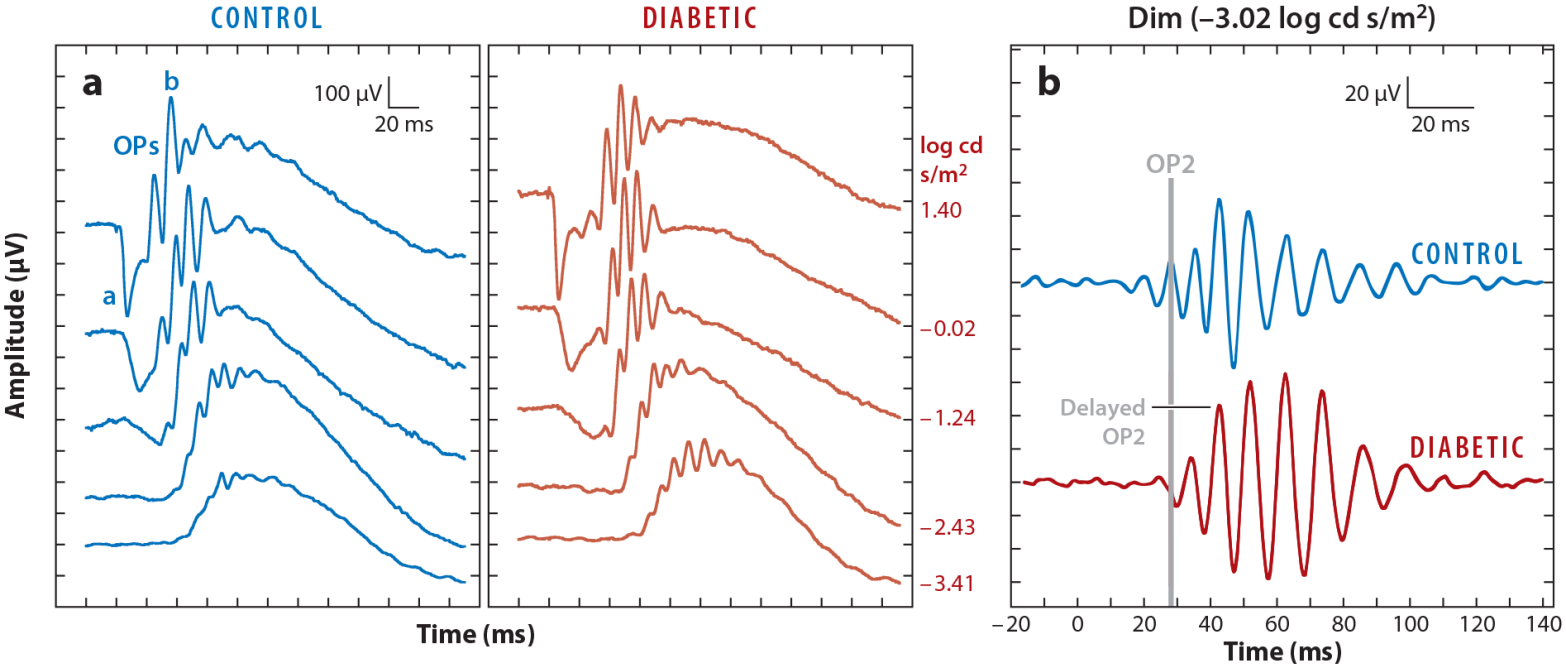
(*a*) Representative raw ERG waveforms of control and diabetic animals at 5 weeks post STZ with a-, b-, and OP waves labeled. No differences were seen in ERG a- and b-wave responses at 5 weeks post STZ. (*b*) Representative OP waveforms from control and diabetic mice in response to a representative dim (–3.02 log cd s/m^2^) flash. Rod-driven OPs had delayed implicit times in diabetic animals. Flash onset was at 0 ms. The gray line indicates OP2 for the control animal. Figure adapted with permission from Kim et al. (2018). Abbreviations: cd, candela; ERG, electroretinogram; OP, oscillatory potential; STZ, streptozotocin.

The ERG measures retinal neuronal activity in response to a light stimulus and has been an in-valuable tool to determine if retinal function is changing early in diabetes. The ERG is measured as the voltage difference between an electrode on the cornea or eye and a reference electrode at a distant site on the head. ERGs can be meaured in vivo in both humans and animal models, so direct comparisons can be made. ERG measurements can also be repeated over time and could potentially be used as a screening mechanism for early retinal dysfunction (Pardue et al. 2014). The full-field ERG has several distinct waves that have been attributed to specific cell types in the retina (Green & Kapousta-Bruneau 1999, Robson & Frishman 1998, Wachtmeister & Dowling 1978). The a-wave represents photoreceptor outer segment activity (Green & Kapousta-Bruneau 1999, Robson & Frishman 1998) (Figure 4). The b-wave represents ON bipolar cell activity that responds to the onset of light (Green & Kapousta-Bruneau 1999, Robson & Frishman 1998). The oscillatory potentials (OPs) ride on the b-wave and represent interactions between bipolar cells and amacrine cells (Green & Kapousta-Bruneau 1999, McCall et al. 2002, Wachtmeister & Dowling 1978). Other specialized ERGs that use different light stimuli, such as the photopic negative response (PhNR); the multifocal ERG (mfERG), which stimulates small portions of the retina (Hood et al. 2002); and the scotopic threshold response (STR), all reflect activity primarily from ganglion cells (Robson & Frishman 1998, Viswanathan et al. 1999).

The most consistent ERG changes seen in human diabetic patients are in the OPs. Many studies reported reduced or delayed OPs in patients with type 1 (Juen & Kieselbach 1990, Parisi et al. 1997) and type 2 (McAnany et al. 2020, Motz et al. 2020, Yoshida et al. 1991) diabetes who had no symptoms of DR. Reduced OP amplitudes in patients without DR are correlated with an increased chance of retinopathy progression (Bresnick & Palta 1987, Simonsen 1980) and decreases in contrast sensitivity (Kawasaki et al. 1986). OP changes developed after 10 years of diabetes (Parisi et al. 1997), and scotopic, i.e., rod-driven, OPs are preferentially affected (Pardue et al. 2014). No changes in a- or b-waves in diabetic patients with no DR have been reported (Kizawa et al. 2006, Vadala et al. 2002), and although cone sensitivity may be reduced (McAnany & Park 2019), that reduction does not correlate with changes in OPs (McAnany et al. 2020).

In rodent models of diabetes, delays in OP implicit times and decreases in OP amplitude appear as early as 3 weeks after diabetes induction with STZ (Aung et al. 2013, Hernandez et al. 2013, Kur et al. 2016, Layton et al. 2007, Li et al. 2020, Piano et al. 2016, Sergeys et al. 2019, Shinoda et al. 2007, Zhang et al. 2011), suggesting early inner retinal damage (Figure 4). Changes are observed earlier in the scotopic rod pathway than in the photopic cone pathway (Pardue et al. 2014, Piano et al. 2016), suggesting that the rod circuits may be preferentially dysfunctional. These changes mirror those in diabetic humans with no DR (Pardue et al. 2014).

Unlike ERG measurements in human diabetic patients with no DR, some studies using rodent models of diabetes observed changes in a-waves at early time points (2–4 weeks after STZ) (Naderi et al. 2019, Piano et al. 2016, Samuels et al. 2015), often in scotopic light levels. Transretinal recordings of rod activity decreased 2 months after STZ (Berkowitz et al. 2015). However, other studies found no changes in a-waves at 4–22 weeks after STZ (Aung et al. 2013, Bui et al. 2009, Kohzaki et al. 2008, Kur et al. 2016, Pardue et al. 2014, Ramsey et al. 2006, Samuels et al. 2012). This suggests that a-waves are an inconsistent measure of diabetic damage. Some rodent model papers also reported decreased amplitudes or increased implicit times of b-waves after 2–4 weeks of diabetes (Aung et al. 2014, Hernandez et al. 2013, Miller et al. 2018, Naderi et al. 2019, Piano et al. 2016, Samuels et al. 2015, Zhang et al. 2011), although others reported no changes after 4–12 weeks (Aung et al. 2013, Kohzaki et al. 2008, Ramsey et al. 2006). A previous study found that the b-wave and OP amplitudes can be related in diabetic rodent models (Layton et al. 2007), and others have reported that changes in inhibition that affect OPs may also affect b-waves (Herrmann et al. 2011, Naarendorp & Sieving 1991, Robson et al. 2004, Smith et al. 2015, Travis et al. 2018). This interrelationship may explain discrepancies between human and rodent b-wave measurements. There can also be significant variations in blood glucose levels among studies using rodent diabetic models, which would be interesting to correlate with changes in ERG waves. However, a systematic review concluded that most papers do not provide raw data for ERG measurements, so the magnitude of effects is difficult to determine (Lelyte et al. 2022).

Changes in the more complex ERG measurements, the PhNR (McFarlane et al. 2012) and mfERG (Bronson-Castain et al. 2012, Dhamdhere et al. 2012, Laron et al. 2012, Tan et al. 2014), are found in human patients with no DR. PhNR differences were correlated with HbA1c and glucose control (McFarlane et al. 2012), and several studies suggested that changes in mfERG were larger in patients with type 2 diabetes (Bronson-Castain et al. 2012, Dhamdhere et al. 2012). Additionally, in studies of diabetic humans with no DR where both mfERGs and vasculature were measured in the same retinal areas, lowered mfERG activity predicted later vasculature growth into that area (Harrison et al. 2011, Ng et al. 2008). Although PhNR and mfERG are not routinely measured in rodent models, there are several reports of reductions in a parallel measurement—the STR—4–12 weeks after STZ (Bui et al. 2009, Kohzaki et al. 2008); however, other studies saw no STR changes (Kaneko et al. 2000, Liu et al. 2020). All of these ERG studies together suggest that the most likely site of early damage in the diabetic retina is in the inner retina.

### Changes in Retinal Neuronal Structure in Early Diabetes

**Figure 5:**
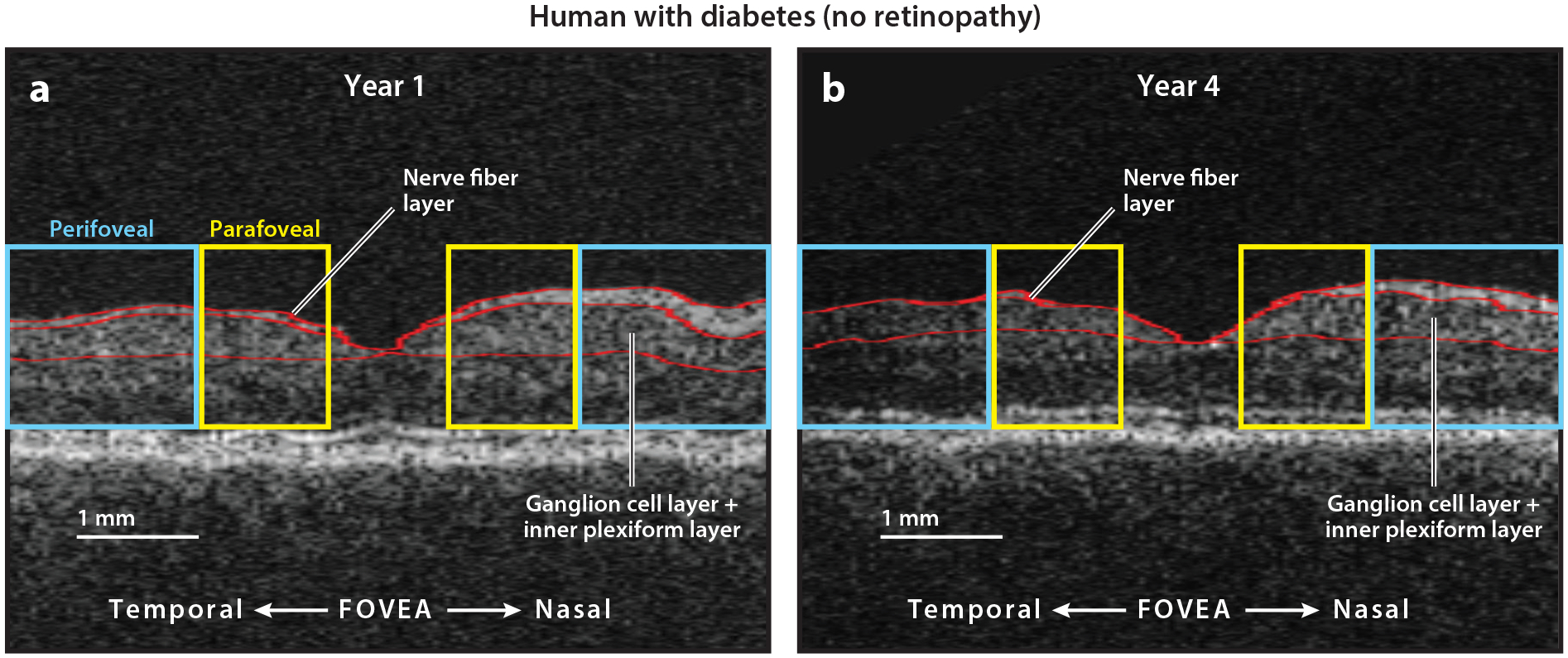
Automated stratus OCT (horizontal B-scans through the fovea) analysis of the right eye of a subject (42-year-old female without DR) (*a*) at the baseline visit and (*b*) at the fourth-year visit showing generalized loss of the NFL and GCL+IPL over this time period in the parafoveal (*yellow outline*) and perifoveal (*light blue outline*) regions. The top red line is the inner limiting membrane that represents the inner boundary of the retina separating the vitreous cavity and the NFL. The middle red line is the boundary between the NFL and the GCL. The bottom red line is the boundary between the IPL and INL. At year 4, the loss of the NFL is so profound in this subject that it is hard to differentiate the top and middle red lines on the temporal (left) side of the fovea. (Scale bar: 1 mm; the width of the scan is 6 mm.) Figure adapted with permission from Sohn et al. (2016). Abbreviations: DR, diabetic retinopathy; GCL, ganglion cell layer; INL, inner nuclear layer; IPL, inner plexiform layer; NFL, nerve fiber layer; OCT, optical coherence tomography.

Functional ERG changes can be due to changes in neuronal activity or loss of neurons of specific types. In the human retina, structural changes in diabetes have been measured with spectral domain optical coherence tomography imaging (Figure 5) that can measure the thicknesses of different retinal layers. Many studies in humans with diabetes but no DR observed a thinner retinal nerve fiber layer that is formed by the axons of ganglion cells going to the optic nerve (Bronson-Castain et al. 2012, Gundogan et al. 2016, Lopes de Faria et al. 2002, Sohn et al. 2016, Verma et al. 2012) and a thinner ganglion cell layer (Karti et al. 2017, Montesano et al. 2021, Pinilla et al. 2020, Sahin et al. 2018, Sohn et al. 2016) than in humans without diabetes.

In animal models of diabetes, TUNEL and/or caspase-3 labeling suggesting the beginning of neuronal apoptosis was reported 2–4 weeks after STZ (El-Remessy et al. 2006, Gastinger et al. 2006, X. Li et al. 2013, Scuderi et al. 2014), primarily in the ganglion cell and inner nuclear layers. Mouse models of diabetes exhibited no loss of rod bipolar cells, horizontal cells, or GABAergic or glycinergic amacrine cells 6–12 weeks after diabetes onset (Hombrebueno et al. 2014, Moore-Dotson et al. 2016). Some studies report ganglion cell layer or labeled ganglion cell loss 4–8 weeks after STZ (Ali et al. 2019, Sergeys et al. 2019, Yang et al. 2012, Zhang et al. 2011), but others saw no cell loss after 6 weeks (Flood et al. 2020, Hombrebueno et al. 2014, Moore-Dotson et al. 2016) or 10 months (Howell et al. 2013). The results from both diabetic humans with no DR and early diabetes animal models again suggest that damage in early diabetes primarily occurs in the inner retina.

## MECHANISMS OF RETINAL NEURONAL DYSFUNCTION IN EARLY DIABETES

Changes to the ERG and visual function can reflect cell death or dysfunction in neuronal activation or connections between neurons. Given the significant early changes that have been observed in diabetic humans without DR and animal models of diabetes, it is important to understand the neuronal and circuit mechanisms of these changes. Signaling in the retina has two main pathways. First is the excitatory pathway, which starts with photoreceptors and relays input to bipolar cells that then send input to ganglion cells, which are the output of the retina (Figure 3). Second are the inhibitory pathways mediated by horizontal cells and amacrine cells, which feedback onto photoreceptors and bipolar cells or ganglion cells, respectively. Photoreceptors and bipolar cells use glutamate as a neurotransmitter, and the majority of amacrine cells use the inhibitory neurotransmitters GABA and glycine (Masland 2012). Changes to synaptic signaling between retinal neurons are suggested by previous reports that synaptophysin and other presynaptic proteins changed expression after 1–3 months of diabetes (VanGuilder et al. 2008).

### Changes in Retinal Neuronal Inhibition in Early Diabetes

**Figure 6:**
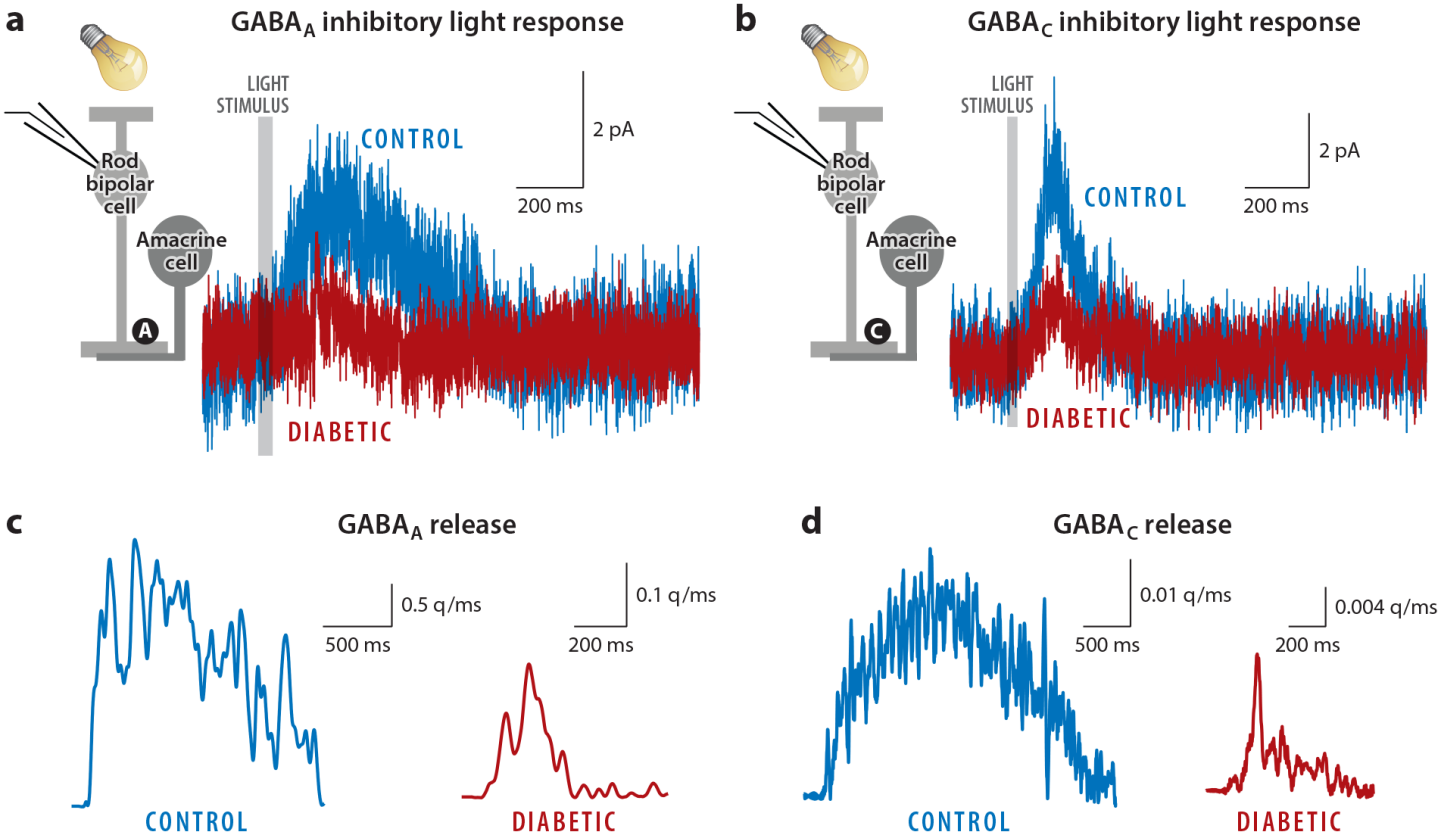
GABAergic inhibition from amacrine cells to rod bipolar cells is decreased after 6 weeks in a mouse model of diabetes. (*a*) GABA_A_ receptor light-evoked inhibitory synaptic currents (L-IPSCs) are reduced in diabetic rod bipolar cells (30 ms light stimulus, 4.75*105 Rh*/rod/sec, *gray bar*). (*b*) L-IPSCs mediated by GABA_C_ receptors are reduced in diabetic rod bipolar cells (30 ms light stimulus, 4.75*105 Rh*/rod/sec). (*c*,*d*) GABA release onto rod bipolar cell (*c*) GABA_A_ and (*d*) GABA_C_ receptors [estimated from deconvolution analysis of GABA receptor–mediated spontaneous (s)IPSCs and L-IPSCs] is reduced in diabetes. Figure adapted with permission from Moore-Dotson et al. (2016).

The prevalent changes in OPs in early diabetes suggest changes in inhibition from amacrine cells, as OPs can be eliminated or changed by modulating levels of inhibitory neurotransmitters or receptors (Green & Kapousta-Bruneau 1999, McCall et al. 2002, Moller & Eysteinsson 2003, Wachtmeister 1980, Wachtmeister & Dowling 1978). While in vivo ERG recordings are useful for holistic and repeatable measurements of the retina, retinal neuronal circuit mechanisms can be studied using acutely isolated retinal preparations in vitro that still respond to light but allow recording from single identified neurons. Prior reports have used this type of preparation to record either light- or electrically evoked inhibition onto the bipolar cells in the rod pathway (rod bipolar cells). In studies using the natural stimulus of light, light-evoked GABAergic inhibition onto bipolar cells in the rod pathway was reduced after 6 weeks of diabetes (Moore-Dotson et al. 2016) (Figure 6). Reduced light-evoked inhibition would correlate with changes in OPs observed early in diabetes. GABA was released onto GABA_A_- and GABA_C_-type GABA receptors on rod bipolar cells, and GABAergic input to both receptor types was reduced. A further study determined that this change in GABA release was specific to GABAergic amacrine cell activity, and not to upstream inputs, as electrically evoked activity directly from GABAergic amacrine cells was also reduced after 6 weeks of diabetes (Moore-Dotson & Eggers 2019).

Changes to synaptic signaling can be due to changes in neurotransmitter release and/or neurotransmitter receptor expression. For reduced GABAergic inhibition, these changes could be due to loss of GABAergic neurons, changes in GABA release, and/or changes in GABA receptors. GABAergic amacrine cells are preserved after 6 weeks of diabetes (Flood et al. 2020). However, several previous reports found reductions in evoked GABA release after 2–8 weeks of diabetes (Baptista et al. 2011, Castilho et al. 2015, Moore-Dotson & Eggers 2019, Moore-Dotson et al. 2016). Reduced GABA release is likely due to reductions in stimulated amacrine cell calcium signaling (Castilho et al. 2015, Moore-Dotson & Eggers 2019), which would lead to decreased evoked GABA release. This is supported by a decrease in expression of GAD, the enzyme responsible for making GABA in neurons (Honda et al. 1998, Ly et al. 2014), and a decrease in VGAT, the transporter responsible for loading GABA into vesicles, in isolated retinal synapses (synaptosomes) after 2 weeks of diabetes (Baptista et al. 2011). All of these changes would lead to the observed decrease in evoked GABA release.

In contrast to decreased evoked GABA release, other reports found increases in the enzyme that produces GABA (Ishikawa et al. 1996) and retinal GABA accumulation (Ishikawa et al. 1996, Ramsey et al. 2006) 8–12 weeks after STZ. This accumulation is supported by increased spontaneous release of GABA observed 6 weeks after STZ, which happened in the same experiments where light-evoked GABA release was reduced (Moore-Dotson et al. 2016). This suggests an imbalance between spontaneous and light-evoked release, which potentially limits evoked release by reducing the number of available vesicles.

However, the effects of diabetes on the GABAergic system may differ between inputs. Increases in release may apply primarily to release onto GABA_A_ receptors, given that two previous studies found either decreased GABA_C_ receptor spontaneous activity (Castilho et al. 2015) or no changes (Moore-Dotson et al. 2016) after 2–6 weeks of diabetes. Increased response of both receptor types has been observed: Increased sensitivity of GABA_C_ receptors was seen after 12 weeks of diabetes (Ramsey et al. 2006), and an increased amplitude of spontaneous GABA_A_ receptor currents was observed after 6 weeks of diabetes (Moore-Dotson et al. 2016). This increased response was not accompanied by changes in GABA_A_ receptor numbers, suggesting that GABA_A_ receptor sensitivity was increased as well. Together, these reports suggest that the primary visual effect of diabetes on retinal inhibition is reduced light-evoked GABA release from amacrine cells, which could explain the smaller amplitudes and delayed implicit times of OPs from ERG measurements.

### Changes in Retinal Neuronal Glutamate Signaling in Early Diabetes

**Figure 7:**
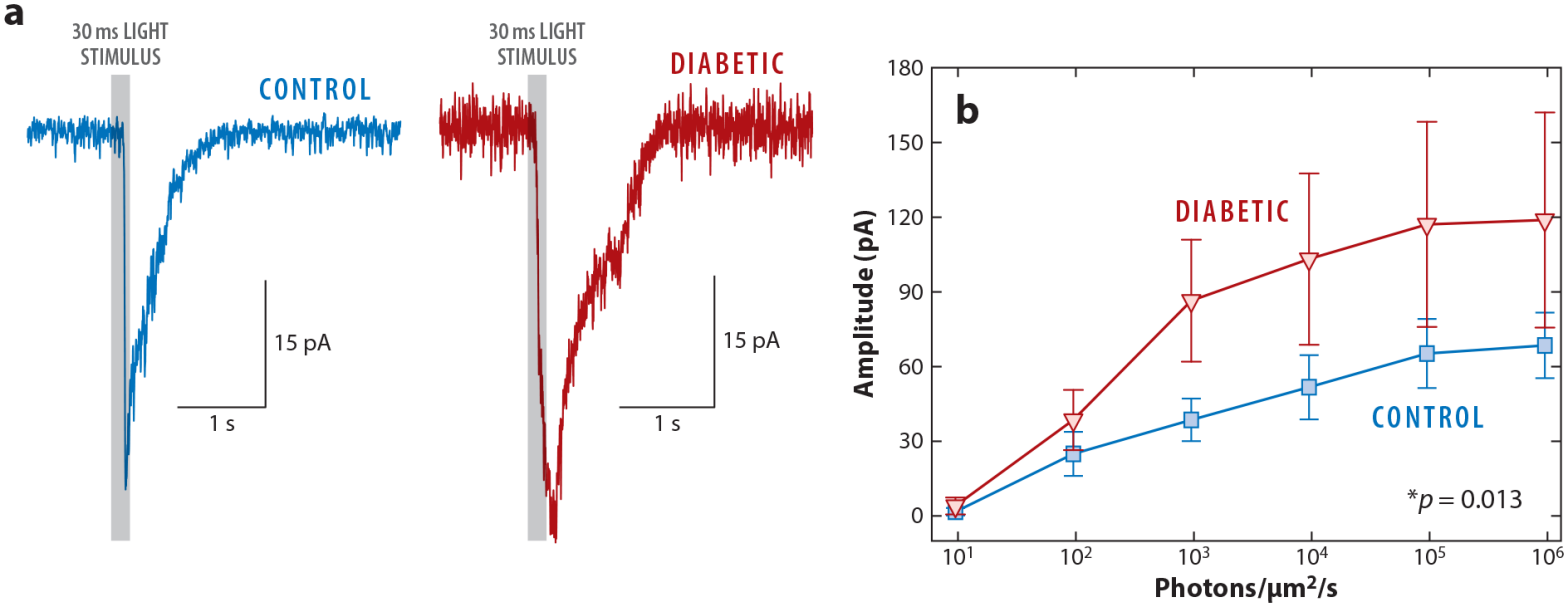
Light-evoked excitation to ON sustained (ON-s) ganglion cells under dark-adapted conditions is increased after 6 weeks of diabetes. (*a*) Example traces of light-evoked responses in a control and diabetic ON-s ganglion cell (gray bars are 30 ms light stimuli). (*b*) Average peak amplitude of light-evoked excitatory postsynaptic currents (EPSCs) from control (*blue*) and diabetic (*red*) ON-s ganglion cells. Control *n* = 14 and diabetic *n* = 19. *p*-Value reported for the main effect of diabetes using a two-way ANOVA and accompanied by * to indicate that it is significant. Figure adapted with permission from Flood et al. (2020).

Changes in b-waves and STRs suggest potential changes in retinal glutamatergic signaling in diabetes. Excitatory rod bipolar cell responses to light did not change after 6 weeks of diabetes (Moore-Dotson et al. 2016), suggesting that b-wave changes could be due to bipolar cell inhibitory input changes, as has been previously reported (Herrmann et al. 2011, Naarendorp & Sieving 1991, Robson et al. 2004, Smith et al. 2015, Travis et al. 2018). However, light-evoked outputs of bipolar cells were increased after 6 weeks of diabetes (Flood et al. 2020, Moore-Dotson et al. 2016) (Figure 7), potentially due to decreased inhibition to bipolar cells. Additionally, many groups found differences in the components of glutamate synaptic transmission in early diabetes. Early diabetes reduced protein or messenger RNA (mRNA) expression of the vesicular glutamate transporters VGlut1 or VGlut2 in the retina (Baptista et al. 2011, Lau et al. 2013, Ly et al. 2014). In contrast, multiple studies found increased retinal glutamate levels after 7–12 weeks of diabetes (Ali et al. 2019, Kowluru et al. 2001, Lieth et al. 1998). This is supported by findings of increases in both light-evoked and spontaneous glutamate release from bipolar cells after 6 weeks of diabetes (Castilho et al. 2015, Flood et al. 2020, Moore-Dotson et al. 2016). Ganglion cells also exhibit increases in spontaneous spiking after 12 weeks of diabetes (Cui et al. 2019, Yu et al. 2013). Increased glutamate receptor levels were found 2–6 weeks after diabetes onset (Lau et al. 2013, Santiago et al. 2009, Semkova et al. 2010). All of these changes collectively suggest that ganglion cells could experience excess glutamatergic input that leads to excitotoxicity in early diabetes (Calvo et al. 2020, Schluter et al. 2020).

### Changes in Retinal Dopaminergic Signaling in Early Diabetes

Dopamine is released by dopaminergic amacrine cells to reduce retinal sensitivity and allow retinal neurons to adapt to increased light levels (Witkovsky 2004). Interestingly, retinal dopamine levels are lower than in control retinas after 3–12 weeks of diabetes (Aung et al. 2014, Lahouaoui et al. 2016, Li et al. 2020, Nishimura & Kuriyama 1985). The retina contains one amacrine cell type—the dopaminergic amacrine cell—that produces and releases dopamine in response to increased light intensities (Mills et al. 2007). The retina also contains three different types of dopamine receptors that respond to this dopamine. D1 dopamine receptors (D1Rs) are part of the D1 G protein–coupled receptor family, which lead to increases in cAMP after dopamine binds (Witkovsky 2004) and are expressed in horizontal cells and subsets of amacrine cells, bipolar cells, and ganglion cells (Farshi et al. 2016, Veruki & Wassle 1996). D2Rs and D4Rs are D2-family dopamine receptors that lead to a decrease in cAMP (Witkovsky 2004) and are localized on dopaminergic amacrine cells (D2) (Derouiche & Asan 1999, H. Li et al. 2013, Veruki 1997) and in photoreceptors and some inner retinal neurons (D4) (Cohen et al. 1992, H. Li et al. 2013). Lowered dopamine levels in the diabetic retina could affect both dopamine release in response to light adaptation and the response of retinal neurons to dopamine via changes in dopamine receptor expression or sensitivity. Lower levels of dopamine in the rodent diabetic retina reduce light-evoked dopamine release (Nishimura & Kuriyama 1985) and light adaptation of a type of ganglion cell (Flood et al. 2020) after 3–6 weeks of diabetes. D4R modulation of light-evoked ganglion cell signaling is also reduced after 6 weeks of diabetes, independent of changes in D4R mRNA expression (Flood et al. 2022). Since the effect of dopamine on retinal neurons is to decrease sensitivity to light, reducing the effects of light adaptation causes retinal ganglion cells to be overexcited after light adaptation. Interestingly, increasing retinal dopamine levels with L-DOPA treatment reduces the effects of diabetes on retinal ERGs, visual acuity, and contrast sensitivity in animal models and humans with diabetes (Aung et al. 2014, Kim et al. 2018, Motz et al. 2020). Together, these results suggest that modulation of dopamine levels or dopamine receptors could be a potential treatment for retinal neuronal dysfunction in diabetes, given the reduced but not absent effect of dopamine in the retina.

### Diabetes May Have Distinct Effects on Retinal Pathways

Retinal signaling can be broken into multiple pathways that signal different aspects of the visual scene. Beginning at the level of the photoreceptors, information is separated into the dim-light, rod-mediated pathway versus the bright-light, cone-mediated pathway. At the level of the bipolar cells, signaling is further broken into cells that respond to the onset of light (ON pathway) and those that respond to the offset of light (OFF pathway). Several groups have reported potential differences in the effects of diabetes on different retinal pathways. Early diabetes has a larger effect in ON ganglion cells. After 3 months of diabetes (STZ), ON, but not OFF, ganglion cells had reduced dendritic field size, decreased capacitance, decreased resting membrane potential, and increased excitability (Cui et al. 2019). After 3 months of diabetes in the Akita mouse model, ON-α ganglion cells, but not OFF, exhibited increased dendrite length (Gastinger et al. 2008). The rod pathway is also significantly affected in early diabetes (Aung et al. 2013, Kohzaki et al. 2008, Liu et al. 2020, Moore-Dotson et al. 2016). Indeed, the effects of diabetes on the rod pathway may occur earlier and be stronger than those on the cone pathway (Pardue et al. 2014). These studies suggest that damage to retinal neurons may be specific not only to cell types, but also to pathways.

### Potential Treatments Related to Neuronal Dysfunction

Current treatments for DR focus on treating vascular dysfunction, which is the late stage of diabetic retinal damage. These treatments include panretinal photocoagulation, which uses a laser to burn peripheral retinal areas to reduce leaky blood vessels and inhibit further vessel growth. This technique can significantly reduce severe DR vision damage but causes peripheral vision loss due to destruction of retinal tissue and can have other complications (Chakravarthy & Devanathan 2018). Current treatments also include anti–vascular endothelial growth factor (VEGF) antibodies (ranibizumab/Lucentis and bevacizumab/Avastin), which inhibit VEGF and improve visual acuity in some DR patients (Brown et al. 2013, Nguyen et al. 2012, Osaadon et al. 2014, Schmidt-Erfurth et al. 2014). However, only 30–40% of DR patients respond to these anti-VEGF treatments (Gonzalez et al. 2016, Yang et al. 2016). Given the complications and incomplete nature of these treatments, it is evident that additional treatments are required. All treatments that focus on vascular factors are necessarily late-stage treatments. It would be ideal to develop neuroprotective treatments that could target early retinal damage.

**Figure 8:**
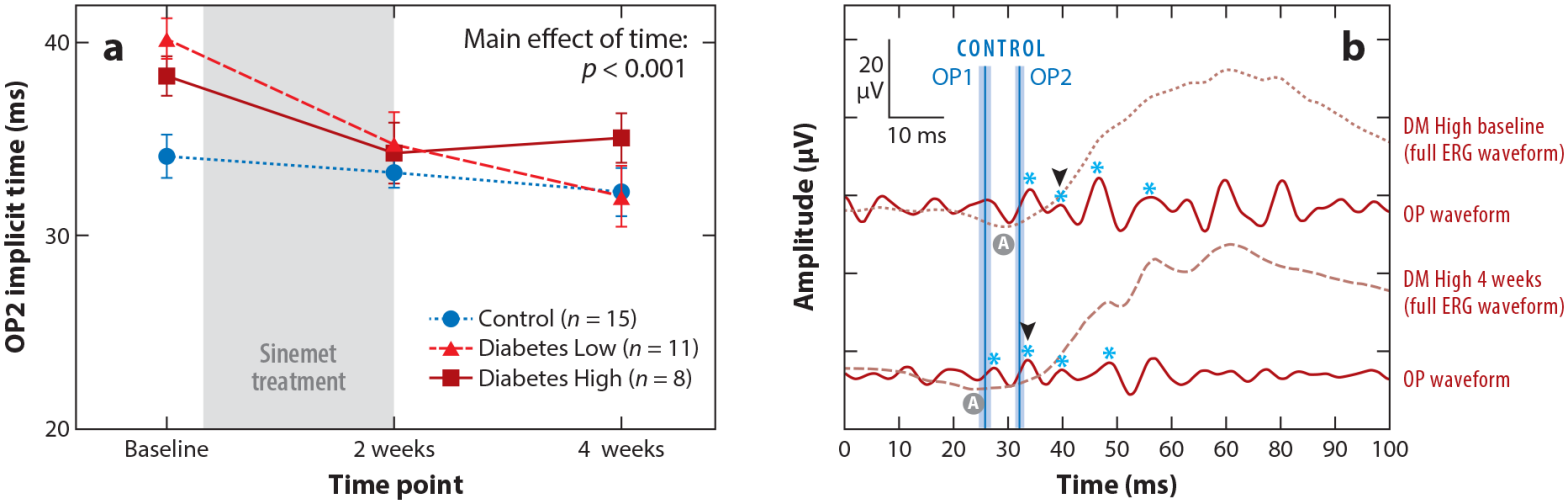
Sinemet (L-DOPA with Carbidopa) treatments improve OP implicit times in eyes in the group with diabetes without retinopathy. (*a*) After 2 weeks of Sinemet treatments, both high- and low-dose groups had OP2 implicit times that were indistinguishable from control subjects. This effect was maintained at 4 weeks, following a 2-week washout period of the drug. (*b*) Representative OP waveforms from a Diabetes High participant at baseline and 4 weeks (2 weeks of Sinemet treatment plus 2-week washout period). The OP waveforms are overlaid with the full ERG. Light blue asterisks indicate the four OP peaks, and black arrowheads indicate OP2 peaks in both DM baseline and DM 4 weeks after treatment. The shifts in the asterisks and arrowheads to the left 4 weeks after treatment indicate improvement in OP implicit time. Figure adapted with permission from Motz et al. (2020). Abbreviations: ERG, electroretinogram; OP, oscillatory potential.

Various potential neuronal targets have been proposed for therapeutic approaches (Barber & Baccouche 2017, Pardue & Allen 2018). This has suggested that DR should be approached as a neurovascular disease (Abcouwer & Gardner 2014, Sundstrom et al. 2018). These treatments typically focus on general neuroprotection or reduction of oxidative stress in neurons and glia (Ou et al. 2022). One potential treatment that has been suggested to specifically modulate neuronal signaling is dopamine, given the low dopamine concentrations in early diabetes (Aung et al. 2014, Lahouaoui et al. 2016, Nishimura & Kuriyama 1985). Dopamine supplementation can reduce retinal ERG changes in early diabetes and changes in visual acuity and contrast sensitivity (Aung et al. 2014, Kim et al. 2018). The visual acuity and contrast sensitivity were also modulated by specific dopamine receptor agonists (Aung et al. 2014). Dopamine supplementation also reversed ERG OP changes in human patients who had diabetes with no DR (Motz et al. 2020) (Figure 8). This is an avenue for future investigation, especially of the effects that dopamine supplementation has on neuronal responses and on early vascular changes, as was shown by a previous study where modulation of G protein–coupled receptors modulated retinal vasculature in diabetes after 8 months of diabetes (Kern et al. 2021). Other treatments that modulate calcium signaling within neurons or limit excitotoxicity would also be good candidates, given the calcium dysfunction and overexcitation in diabetic retinal neurons discussed above.

## SUMMARY

It is clear that diabetes causes early changes in retinal neuronal and visual function, prior to any clinical symptoms of DR in human diabetic patients. These early changes, especially those due to amacrine and ganglion cells in the inner retina, such as ERG OPs and contrast sensitivity, can be replicated in rodent models of diabetes after only a few weeks of diabetes. This suggests that mechanisms of diabetic neuronal damage found in rodent models, such as reduced GABA release and increased glutamate signaling that may lead to excitotoxicity, may be relevant for treatment of human disease. At least one treatment that directly modifies neuronal activity with dopamine supplementation has been tested in animal models of diabetes and humans with no DR and found to limit or reverse changes in retinal function measured by ERG and visual function measurements. As dopamine modulation in a rodent model has also led to reductions in early vascular changes in diabetes, this suggests that understanding and modifying neuronal dysfunction may lead to early diabetes treatments.
